# Oxygen impact and reactivity trials: A new perspective on emergency response precautions

**DOI:** 10.1016/j.heliyon.2023.e14474

**Published:** 2023-03-11

**Authors:** Andrew Byrnes, Clayton Rawson, Brian Patchett, Daniel DeMille, Merrill Halling

**Affiliations:** a Utah Valley University, Emergency Services, 3131 Mike Jense Parkway, Provo, UT 84601, USA; b Utah Valley University, Chemistry, 800 W. University Parkway, Orem, UT 84058, USA; c Utah Valley University, Physics, 800 W. University Parkway, Orem, UT 84058, USA; d Utah Valley University, Utah Fire and Rescue Academy, 3131 Mike Jense Parkway, Provo, UT 84601, USA

**Keywords:** impact Pressure, Adiabatic, Asphalt, Combustible materials, Ignition, Combustion, Emergency response, Utah valley university, UVU, LOx

## Abstract

The objective of this research was to verify and qualify what has been traditionally taught as fact during first responder's hazardous materials training regarding response precautions to and the likely behaviors of liquid oxygen (LOx) during a release. Subject matter experts disagreed that these precautions were well-founded in precedent or science. Findings showed that impact pressure causes a reaction in LOx and asphalt under specific conditions. These conditions are not realistic during an emergency response. No reactions were observed by combining LOx with common saturated and unsaturated hydrocarbons and alcohols. No reactions were observed driving fire apparatus through a LOx pool on asphalt. No reactions were observed by combining LOx and combustible materials. No reactions were observed when spark ignition was used as a source for combustion. Pilot ignition sources were introduced directly into a LOx pool on asphalt without a significant reaction. Immediate and violent reactions were observed when pilot ignition or arc ignition was used to initiate combustion when combustible materials were in an ultra-high gaseous or liquid oxygen environment. Without flaming or arc ignition sources, no reactions were observed.

## Highlights

• Emergency responders are trained in untested procedures for LOx incidents.
• LOx-soaked and frosted over asphalt will not react to impact pressure alone.
• In combination with LOx, hydrocarbons, and combustible materials fail to react.
• Without a flaming or arcing ignition source, reactions are not observed.
• Adiabatic compression of oxygen bubbles against a rigid barrier causes reactions.

## Graphical abstract

**Figure:**
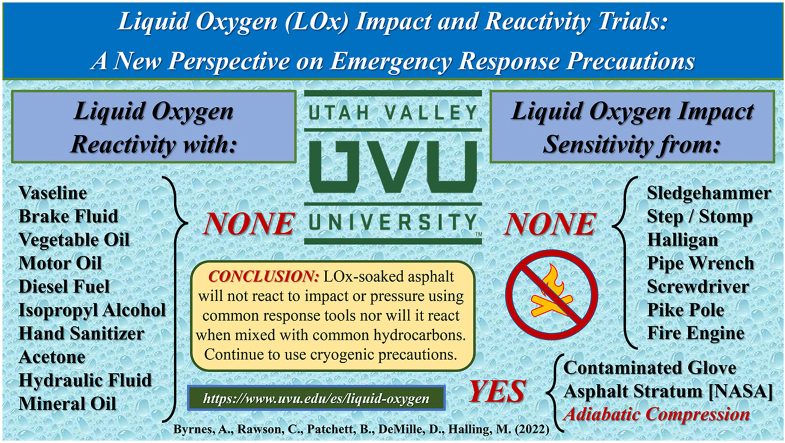

## 1 Introduction

For decades, hazardous materials instructors have taught as fact the following guidelines and procedures for handling emergencies involving the accidental or intentional release of liquid oxygen (LOx). First, do not step on the frozen asphalt because it may explode; do not drive over the frozen asphalt because the weight of the vehicle could cause a detonation; do not allow LOx to contact combustible materials, such as dried grass in the median or hydrocarbons such as diesel fuel, because it will ignite spontaneously resulting in a fast and intense fire; finally, wait 30 min after the last frost is gone before stepping on the asphalt due to a potential explosive reaction. The objectives of this research were to test these theories to verify if the presence of LOx, under common emergency response conditions, increases the danger to first responders and limits the efficiency of the emergency response.

LOx response precautions are based on traditional guidance and commonly accepted as accurate. Hazardous materials subject matter experts (SME's) and instructors with a background in hazardous materials emergency response and chemistry were skeptical of these procedures and could not find information indicating procedures had ever been scientifically tested or verified. Understanding that oxygen is a powerful oxidizer, the authors wanted to conduct a series of reactivity tests involving LOx. Reactivity, in this context, is meant to mean both reaction to mechanical impact or pressure, spark and arc ignition, and chemical reaction between LOx and common combustible materials and hydrocarbon fuels. Without testing these theories, hazardous materials instructors are simply passing on unsubstantiated conventional guidance in training.

A comprehensive literature search of the National Emergency Training Library at the National Fire Academy in Emmitsburg, Maryland, revealed none of the precautions regarding LOx emergency response are substantiated or independently verified. Most results were anecdotal and ambiguous, such as this account in Martel's *Chemical Risk Analysis:* “George Claude was seriously injured in 1903 after inserting a candle into liquid oxygen” [1]. The National Fire Protection Association (NFPA) 53, *Recommended Practice on Materials, Equipment, and Systems Used in Oxygen-Enriched Atmospheres*, Annex D, lists many types of LOx incidents but with the caveat “NFPA cannot guarantee the accuracy of the reports.” [2] None of the 63 “incidents” in NFPA 53, Annex D, could be corroborated. Many LOx vendors have produced Safety Data Sheets with vague cautions that say, for example, “LOx will violently oxidize organic material.” The Compressed Gas Association pamphlet 2.7, *Guideline for the Safe Storage, Handling, and Use of Small Portable Liquid Oxygen Systems in Health Care Facilities* states, “Stepping on or rolling equipment across a liquid oxygen spill can result in explosive ignition of combustibles.” [3].

In 2021, emergency responder students attending hazardous materials courses at the National Fire Academy were polled regarding personal experience with any of these phenomena. Of the approximately 30 students polled, all with hazardous materials emergency response experience, none had witnessed any of the phenomenon personally while a majority were familiar with the traditional LOx procedures taught to hazardous materials responders. Further research efforts could not identify a single verifiable incident. Researchers conducted field trials at Utah Valley University (UVU) in Provo, Utah, to test and verify the aforementioned ‘dangers’ of LOx.

To better understand liquid and gaseous oxygen environments, field testing of LOx-soaked asphalt and mechanical impact on runway materials was designed and conducted by NASA in 1973 [4]. NASA fabricated a test apparatus that consisted of four “plummets,” each held by a pin that could be removed remotely via a lanyard. Each plummet weighed 9.07 kg and was 7.5 cm in diameter. The tip of each plummet was 1.27 cm in diameter and 3.8 cm long. Plummets were raised and then pinned at the desired height inside a tube. When the pin was pulled, the plummet slid down the tube to the target. Tubes extended to within 15 cm of the surface. No mention is made in the report regarding the height of the plummet drop. The target area consisted of crumbled asphalt pavement in a 2 m^2^ area approximately 5 cm thick. The tested strata were composed of 2.5 cm of crumbled asphalt on which a 2.5 cm thick solid aluminum block was placed, covered by an additional 2.5 cm of crumbled asphalt, then immersed in LOx. A small asphalt curb was constructed to hold the test stratum and a quantity of LOx inside the test area. Flexible cryogenic tubing perforated along the bottom delivered “10 min of flow” of LOx to the test area surface [4].

NASA's mechanical impact testing on LOx-soaked asphalt resulted in a detonation that blew the test apparatus 30 m into the air and created a debris field 50 m in diameter. UVU researchers wanted to replicate and verify NASA's 1973 test results using a scientific method. NASA used greater quantities of LOx and a larger test area containing the asphalt strata (∼500 cm^2^) which may account for the magnitude of the explosion. UVU trials consisted of a steel cup with an inside area of 182.41 cm^2^, 63% smaller than NASA's test area. (See Fig. 4).

## 2 Materials and methods

**Fig. 1:**
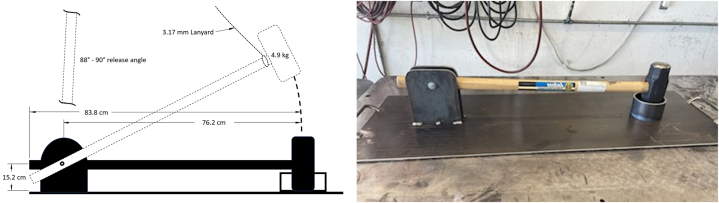
UVU Impact hammer test apparatus [4.9 kg]. (a) Diagram. (b) Photo.

Several asphalt samples measuring approximately 9 cm × 9 cm x 7.5 cm were weighed, immersed in a LOx bath to condition for 30 min, and then weighed again. Samples absorbed varying amounts of LOx measuring between 100 and 1000 ml measured immediately after removal from the LOx bath. These samples were then subjected to a series of impact tests which raised an object to a specified height and dropped it onto the sample. Samples were also struck with a fabricated impact hammer test apparatus designed to replicate a deliberate sledgehammer strike on a LOx-soaked test surface, as depicted in Fig. 1 (a, b).

One of the main objectives of UVU's research was to measure the reactivity of asphalt exposed to LOx in relationship to first responder actions. Drop heights and striking-surface orientations were determined by replicating what may happen if a responder dropped a tool from shoulder height or accidently struck a LOx-soaked surface with the point of a tool. Before the impact tests were performed using LOx, they were repeated a minimum of five times on a Vernier® (Beaverton, OR USA) model FP-BTA Force Plate instrument to acquire initial force and pressure data. The objects that were dropped were selected based on common tools used by first responders. (See Table 1).

**Table 1:** Initial force and pressure values.

Object	Force (kg)	Pressure (kPa)	KE (J)	Drop H (m)	Area (cm^2^)
Hammer	197.1	6779.6	36.88	0.8	2.8
Stepping	85.0	41.4	47.05	0.6	200
Stomping	363.7	460.6	94.09	0.9	77.4
Halligan	161.6	1091.4	45.7	0.9	14.5
Pipe Wrench	164.5	80668.7^a^	13.14	1.2	0.2
Screwdriver	6.8	5274.5	2.09	2.1	0.12
Pike Pole	206.6	103220.7^a^	134.42	1.8	0.12
Fire Engine	2766.9	499.9^a^	13 774.8	N/A	542.9
NASA test	99.7	7718.0	97.55	1.1	1.3

Note: Temperature, barometric pressure, and relative humidity had no bearing on these values.

a These pressures seem high, and the fire engine seems low due to the surface area contact in relationship to the weight of the object.

**Fig. 2:**
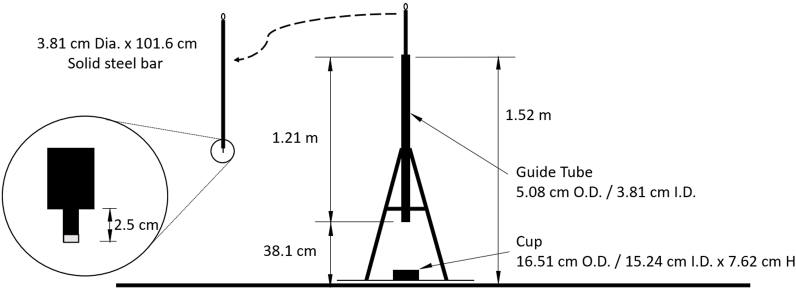
UVU impact apparatus with stainless steel striker pin per ASTM specifications [5].

In 2017, the American Society of Testing and Materials (ASTM) created the *Standard Test Method for Determining Ignition Sensitivity of Materials to Mechanical Impact in Ambient Liquid Oxygen and Pressurized Liquid and Gaseous Oxygen Environments* (G86-17) [5]. ASTM codified the configuration of impact mechanisms used to test the sensitivity of materials to mechanical impact pressure. The apparatus designed and built at UVU was designed in compliance with the ASTM impact testing configuration. (See Fig. 2). The ASTM G86-17 was applied as the standard for impact testing devices. The authors and UVU support the use of this standard for any related future impact testing.

Recordings of the reactions were captured using three high speed cameras: (RED® Epic Dragon, GoPro® Hero 3, and a Phantom® VEO 1310 L. A Sony® NX5U was used to capture real-time video and an additional high speed infrared thermal camera, courtesy of Teledyne FLIR®, was used to detect any thermal changes. These provided outstanding resolution and captured what the naked eye could not see due to the speed of the reaction. In real time, it could be determined that a reaction had occurred by an audible sound similar to a gunshot. These audible reactions were heard by the research team and captured on video, however, sound level data was not captured using audio measuring devices.

**Fig. 3:**
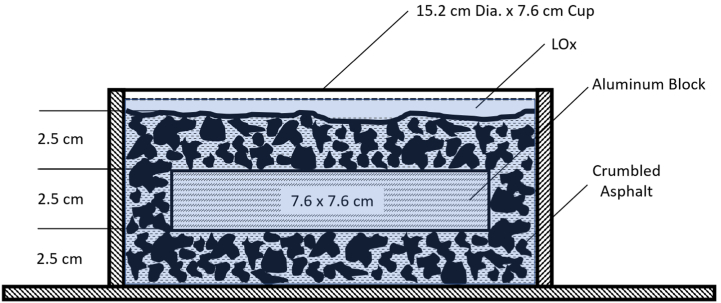
UVU replication of the NASA asphalt test stratum.

UVU researchers tested several control stratum configurations and observed that a reaction would only occur if the unique NASA stratum consisting of a solid aluminum block resting on and covered by crumbled asphalt was used. (See Table 2 and Fig. 3).

**Table 2:** Test stratum variations and reactivity.

Stratum Variation	Result in Reaction
Bottom Layer: Crumbled Asphalt	Yes
Middle Layer: Aluminum Block	
Top Layer: Crumbled Asphalt	
Bottom Layer: Aluminum Block	No
Top Layer: Crumbled Asphalt	
Crumbled Asphalt Only	No
Aluminum Block Only	No

**Fig. 4:**
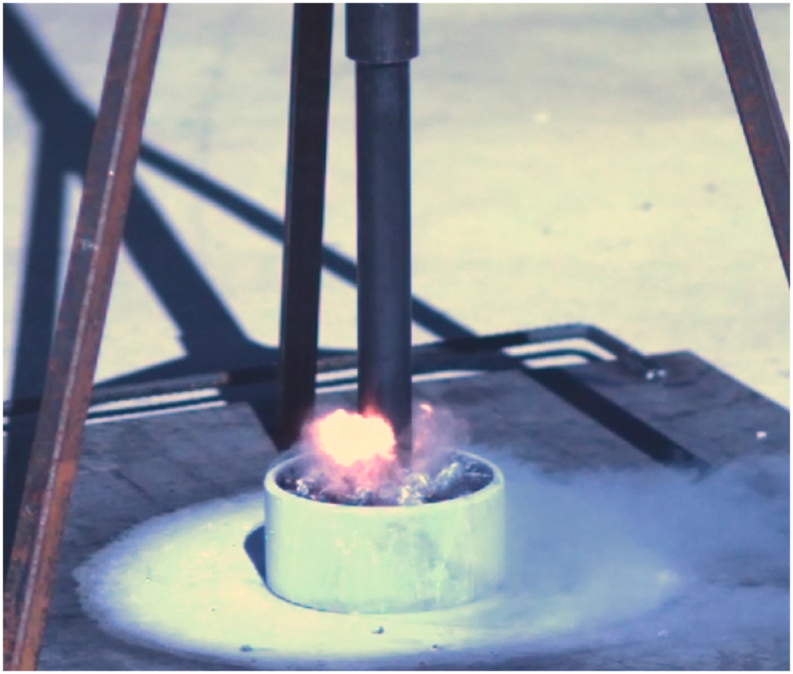
A typical detonation caught on high-speed camera.

Another Objective of this research project was to investigate spontaneous reactions between various hydrocarbon compounds and LOx. Existing hypotheses suggest LOx is such a highly reactive oxidizer that it will react spontaneously with certain fuels. Eleven common hydrocarbon compounds were analyzed by placing 236.5 ml of the hydrocarbon into a Styrofoam cup with an equal amount of LOx. (See Table 3). Each sample was then examined for 1 min for any sign of a chemical reaction followed by another observation at 90 min.

**Table 3:** Reactivity of common hydrocarbons.

Material	July 21^a^	Oct. 21^b^	Mar. 22^c^	Reaction	Flash point F° C°	Vapor Pressure mm/Hg
Vaseline	X	X		none	no data	no data
Brake Fluid DOT 3	X	X		none	>203 > 118	<.01
Vegetable Oil	X	X		none	>625 > 329	no data
Motor Oil 10W30	X	X		none	345 174	<.01
Diesel Fuel	X	X		none	>100 > 38	<1.0
91% Isopropyl Alcohol		X		none	57 13.8	45
Hand Sanitizer			X	none	78 27	no data
Acetone			X	none	0–18	185
AW-46 Hyd. Fluid			X	none	>280 > 138	no data
Mineral Oil			X	none	>234 > 112	no data
Synthetic Motor Oil			X	none	482 250	no data

a July 2021 Temp: 83.5 °F, BP: 30.2, RH: 46%.

b Oct. 2021 Temp: 60.9 °F, BP: 30.4, RH: 48%.

c Mar. 2022 Temp: 32 °F, BP: 25.6, RH: 64%.

Alternate pilot ignition sources, those with a spark, flame, or heat, were tested. A standard highway road flare was introduced into a pool of LOx on a conditioned asphalt surface to determine any spontaneous reaction. A propane handheld torch flame was directed onto an asphalt sample that had been soaked in motor oil and then conditioned under LOx for 30 min. The flame was directed onto the sample for a total of 60 s before being removed for observation of ignition.

Alternative, electrical sources for ignition were tested due to some anecdotal incidents where static electricity was the apparent ignition source. Static electricity and electrical arcing were used as potential sources of igniting LOx-soaked asphalt and carpet and pad configurations. A static charge created by an automotive coil and a 12-V battery system produced a 30 000-V source for an electrical spark across a 3 mm gap between small gauge steel wires. Researchers created an electrical arc by connecting the positive and negative sides of a 12-V battery resulting in arcing of steel rods as a potential ignition source.

## 3 Results and discussion

UVU successfully replicated the 1973 NASA results, with detonations, validating their results [4]. From these tests, researchers concluded that mechanical impact on LOx can cause a reaction to occur using the NASA stratum configuration. It was this configuration of strata that exploded during the NASA test in 1973 and produced detonations at UVU in 2021. ASTM G86-17 standard states that any one reaction in 20 drops of the mechanism (5%) indicates the tested material is “reactive.” [5] UVU researchers observed five reactions in 20 drops of the plummet (25%) and were able to duplicate those reactions again 60 days later. (See Fig. 4).

The drop height for each test was 110 cm as per ASTM G86-17. The force of the impact was 99.7 kg and the pressure was 7722 kPa. Only when the standard variation of the NASA stratum is impact-tested are there consistent positive reactions.

**Fig. 5:**
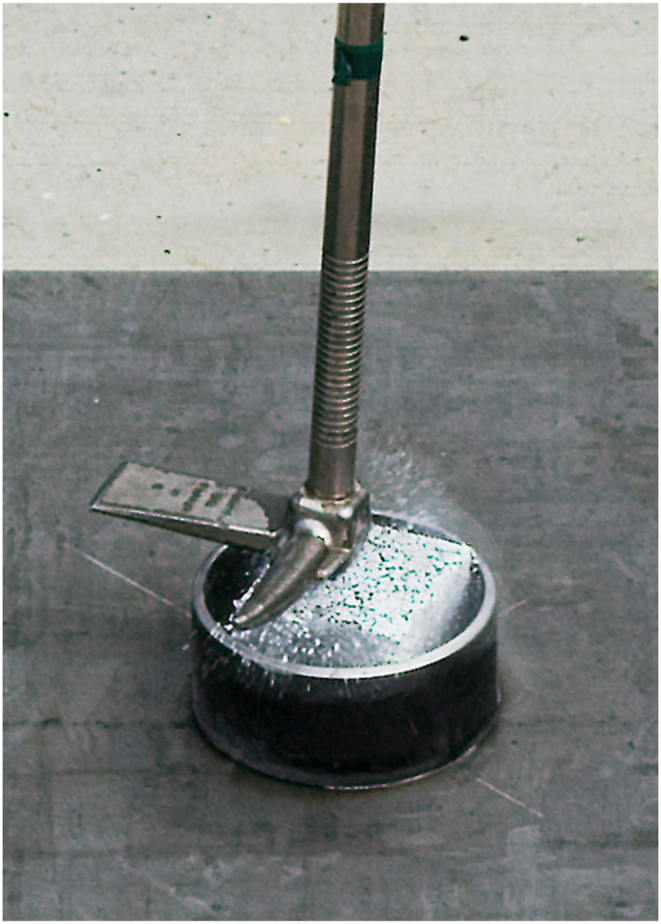
Moment of impact of a Halligan tool dropped on a conditioned asphalt sample.

To provide a more realistic test stratum, UVU used solid asphalt samples, conditioned by soaking the sample in LOx, for further impact testing. The first simulated drop onto a solid asphalt sample was a sand-weighted rubber firefighter's boot (10.9 kg) dropped from .46 m to 0.91 m to simulate an 85 kg person stepping and stomping, respectively, on asphalt samples exposed to LOx. Next, the LOx saturated samples were struck with a 4.53 kg sledgehammer, a 5.1 kg halligan tool (blunt headfirst), a 1.1 kg pipe wrench (headfirst), a 7.5 kg pike pole (point first), and a 0.1 kg cross-point screwdriver (point first). Additionally, the right front tire of a fire engine, with a weight of 2767 kg, drove over a larger conditioned asphalt surface and liquid pool of LOx. After a series consisting of five impact drop tests with each tool and the engine rolled over the LOX pool five times, none of the sources of mechanical impact or pressure caused any reaction in the asphalt. Upon impact, researchers observed only the results of mechanical force on the sample. A typical observation is shown in Fig. 5. It is the opinion of the authors, based on observed reactions, that five trials were sufficient evidence to support the results and the conclusions in section 1.4 of this paper.

**Fig. 6:**
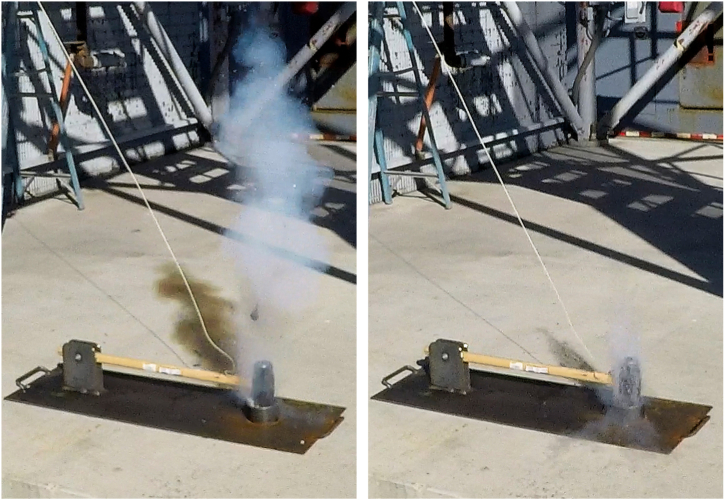
UVU Impact hammer results on the contaminated glove (a) and new glove (b).

Researchers also subjected other materials to impact testing with the impact hammer apparatus, such as a diesel fuel and LOx mixture, LOx-soaked new carpet pad and carpet in their standard configuration, and both new and contaminated NFPA firefighter structural gloves. No reactions were observed except for the glove, which is primarily contaminated with smoke particulate consisting of polycyclic aromatic hydrocarbons (soot) on the surface of the leather. These results were replicated twice over the period of the trials. New leather glove material, with liner, was impacted a total of six times with no observable reactions while the contaminated leather glove with liner was impacted a total of eight times resulting in four detonations (50%). Shown in Fig. 6 (a, b).

**Fig. 7:**
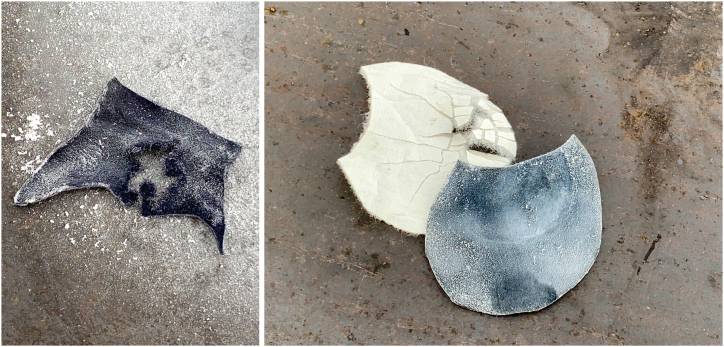
Post-impact: contaminated glove (a) and new glove (b).

The impact testing of the new versus the contaminated firefighter glove proved to be an unrealistic scenario in a real-world response. However, this may be worthy of future testing of standard first responder Personal Protective Equipment (PPE) subjected to LOx exposures. An impactful research objective would be the basic safety of “clean” versus “dirty” first responder PPE to include gloves, boots, and protective gear, which is the typical PPE for a cryogenic response. Post-impact photos of the contaminated glove material and the new glove material are shown in Fig. 7 (a, b).

To determine the non-impact reactivity of LOx, it was poured directly into 11 different hydrocarbon compounds commonly available. Researchers observed no reaction other than freezing the liquid. Saturated and unsaturated hydrocarbons, synthetic and natural compounds, alcohol-based products, various viscosities, and three liquids with flash points below 37.7 °C resulted in no observable reactions other than freezing when in contact with LOx. (See Table 3). Each of these compound mixtures with LOx were observed for 1 min. They were observed again at 90 min with no reaction other than thawing of the mixture. Likewise, combustible materials, such as a cup of potato chips, oily and organic, did not react in contact with LOx. When any of these combinations of hydrocarbon liquids or organic materials and LOx met with an ignition source, the combustion was rapid and intense. Combustion, influenced by LOx, was noticeably more rapid and vigorous than “normal” combustion occurring in atmospheric air at 20.9% oxygen [6].

Researchers recognized the ubiquitous use of highway road flares and their potential as an ignition source during a LOx incident on asphalt. To test the reactivity and sensitivity of LOx and highway flares, LOx was poured directly onto an asphalt surface and a highway road flare was dropped into the pool of liquid oxygen. Other than a marginal increase in flame length on the flare and burning of the flare paper, no additional flaming combustion or explosion occurred. A propane fueled handheld torch was used as a worst-case pilot ignition source on an asphalt sample that had been soaked in motor oil and then conditioned for 30 min under LOx. After 60 s, the flame was taken away from the sample resulting in the immediate extinguishment of the flame. The motor oil could be seen boiling on top of the asphalt block and a small, short-lived, amount of smoke was produced. The flaming combustion was at first vigorous and quickly vaporized the LOx on the sample.

**Fig. 8:**
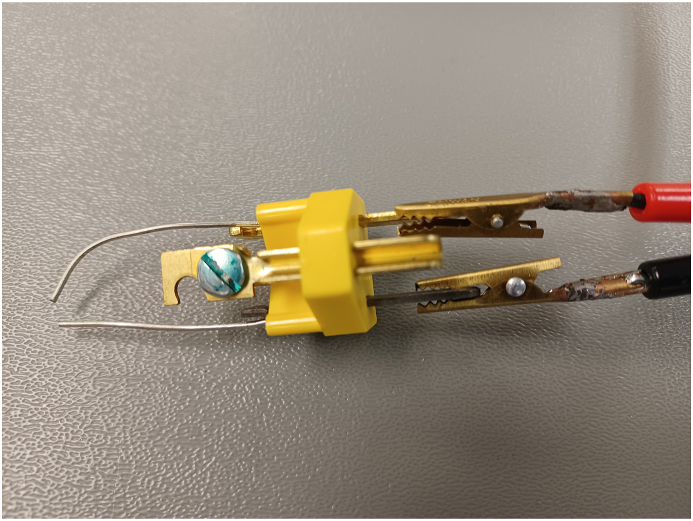
Spark mechanism ignition source using a 3.1 mm gap.

**Fig. 9:**
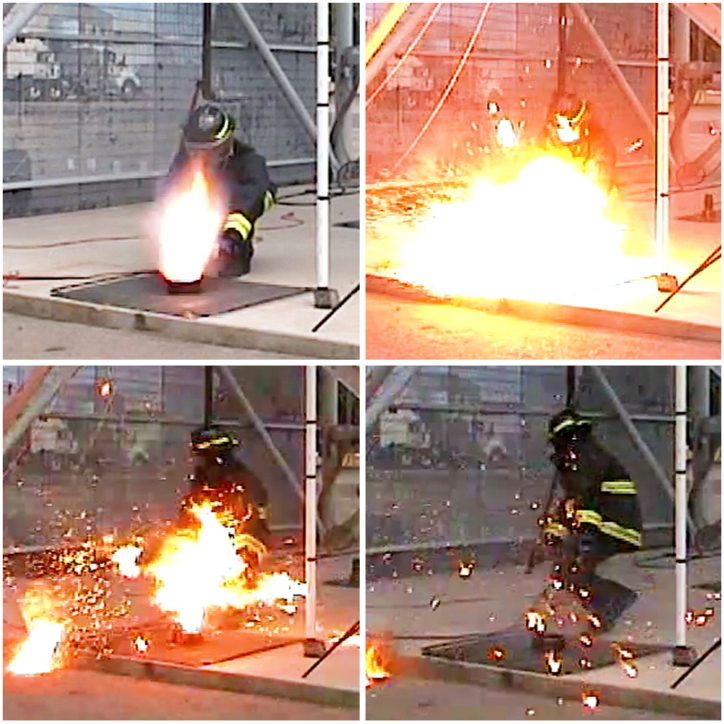
Ignition and flashing of carpet and pad using arc ignition. Photos depict a sequence of events. (Photo credit: Eugene Ngai).

The UVU tests also considered static electricity and arcing as potential ignition sources. Researchers observed that a static spark was an unreliable source of ignition. The spark was hot enough (+982 °C), however, the duration of the heat source, only milliseconds, may have been too brief to cause ignition. (See Fig. 8). Arcing, initiated by connecting the positive and negative sides of a 12 V battery, produced visible sparks and hot molten metal beads which immediately ignited any combustible fuel combined with LOx but would not ignite a solid asphalt sample exposed to LOx. Fig. 9 shows the ignition sequence, using an arc, of LOx-soaked carpet and pad in a normal configuration.

The nature of the experiments conducted at UVU involved several fundamental thermodynamic processes. In particular, adiabatic processes. In an adiabatic process, heat from a physical process is unable to transfer into or out of the system but a key component is that the temperature can, and often does, fluctuate dramatically during the process, it simply does not leave the system. Typically, this is due to the temporal nature of the process, meaning that it is happening so fast that the heat generated is reabsorbed immediately by the system. Fundamental to the process are the heat capacities of the system (C) where the volume of a gas is held constant and the internal pressure is increased $\left(C_{V}\right)$, and a condition under which the pressure remains the same, but the volume is decreased $\left(C_{P}\right)$. An adiabatic process typically involves the ratio of these two terms and is defined through derivation as the value $\gamma =C_{P}/\;C_{V}$. When the concept is applied to the first law of thermodynamics it can be shown that the change in internal energy of a system $\left({dE}_{\mathrm{int}}\right)$ is directly proportional to the pressure, (p), times the change in volume (dV).

dE_int_ = C_v_ ∙ n ∙ dT = - p ∙ dV

Where dT is the change in internal temperature, the heat capacity at constant volume of the gas in question is $C_{V}$, and the number of moles of gas contained in the process is n. This shows that as the volume of a system decreases (meaning that dV is negative) the pressure will increase, causing both the internal energy and the internal temperature to increase. The adiabatic process has been shown, in oxygen rich environments, to generate temperature increases sufficient enough to cause ignition of the gas [7]. Additionally, the compression of collapsing bubbles contained within a liquid has been shown to increase internal temperatures and create brief flashes of light emitted by the plasma induced in the process [8].

Prior work has shown that the adiabatic compression of gases trapped in a combustible liquid can lead to ignition of that gas [[9], [10], [11], [12]]. From this set of experimental trials, the authors’ hypothesis is that the adiabatic compression of the oxygen bubbles, occurring when the striking pin impacted the crumbled asphalt strata with the aluminum block (acting as a rigid boundary) produces temperature levels high enough to create an ignition source during impact testing.

Detonation occurred as the plummet rapidly compressed the micro-bubbles of oxygen gas between the striking pin and the aluminum block as well as the head of the hammer and surface of the contaminated glove. The bubbles of oxygen gas are trapped, instantaneously increasing the pressure and the temperature of the oxygen gas inside the bubble adiabatically. The heat generated serves as the ignition source for the hydrocarbons in the samples and the surrounding enriched oxygen environment.

## 4 Conclusion

This study has shown LOx-soaked and frosted-over asphalt will not react from the pressure associated with being stepped on, stomped on, driven over, or impacted by common response tools that are dropped on it, or even the pressure from a direct sledgehammer strike. LOx, spilled on asphalt, would be extremely difficult to ignite with common ignition sources found on the emergency scene. Heat sources added to LOx/asphalt combinations only increased the rate of vaporization of the LOx without ignition. LOx will not react on contact with common combustibles, organic materials, flammable materials, flammable liquids, and other common hydrocarbons *unless an ignition source is introduced* – in which case the combustion will be violent and instantaneous.

Using the ASTM G86-17 standard, the NASA explosion from the 1973 plummet test was successfully replicated in this study. However, circumstances leading to the detonation of the LOx and asphalt configuration are unrealistic, i.e., the aluminum plate inserted in a crumbled asphalt stratum. Explosions could not be replicated using solid or crumbled asphalt and LOx alone, a much more likely configuration on the scene of an emergency.

Every attempt was made to create and maintain scientific relevance; however, a limitation of the research is that these tests were done to simulate practical environments without the controls that a laboratory provides. The objective was to test LOx, not gaseous oxygen in high concentrations. It is already established that the higher the oxygen concentration of the atmosphere, the more complete the oxidation/reduction reaction would be, resulting in more rapid and complete combustion [6].

Practical testing of additional hazards associated with LOx will be expanded in the future.

Responders should take every precaution necessary when dealing with the primary hazards of LOx, namely embrittlement of surfaces in contact with the super-cooled liquid, high expansion ratios, and frost formations. Anytime LOx is mingled with combustible materials or flammable and combustible liquids, sources of ignition should be eliminated due to the possibility of extremely vigorous combustion. Any modifications to a response agency's procedures should be evaluated carefully based on these conclusions.

## Author contribution statements

**Andrew Byrnes:** Conceived and designed the experiments; performed the experiments; Analyzed and interpreted the data; Wrote the paper.

**Clayton Rawson:** Performed the experiments; Analyzed and interpreted the data; Contributed reagents, materials, analysis tools or data.

**Brian Patchett:** Performed the experiments; Analyzed and interpreted the data; Contributed reagents, materials, analysis tools or data; Wrote the paper.

**Daniel DeMille:** Contributed reagents, materials, analysis tools or data.

**Merrill Halling:** Conceived and designed the experiments; Performed the experiments; Analyzed and interpreted the data; Contributed reagents, materials, analysis tools or data; Wrote the paper.

## Funding statement

Clayton Rawson was supported by 10.13039/100019317Utah Valley University [SHS017].

## Data availability statement

Data included in article/supplemental material/referenced in article.

## Declaration of Interest's statement

The authors declare that they have no known competing financial interests or personal relationships that could have appeared to influence the work reported in this paper.
